# Exploration of natural red-shifted rhodopsins using a machine learning-based Bayesian experimental design

**DOI:** 10.1038/s42003-021-01878-9

**Published:** 2021-03-19

**Authors:** Keiichi Inoue, Masayuki Karasuyama, Ryoko Nakamura, Masae Konno, Daichi Yamada, Kentaro Mannen, Takashi Nagata, Yu Inatsu, Hiromu Yawo, Kei Yura, Oded Béjà, Hideki Kandori, Ichiro Takeuchi

**Affiliations:** 1 The Institute for Solid State Physics, The University of Tokyo, Kashiwa, Japan; 2 RIKEN Center for Advanced Intelligence Project, Tokyo, Japan; 3 Department of Life Science and Applied Chemistry, Nagoya Institute of Technology, Nagoya, Japan; 4 OptoBioTechnology Research Center, Nagoya Institute of Technology, Nagoya, Japan; 5 PRESTO, Japan Science and Technology Agency, Kawaguchi, Japan; 6 Department of Computer Science, Nagoya Institute of Technology, Nagoya, Japan; 7 Graduate School of Humanities and Sciences, Ochanomizu University, Tokyo, Japan; 8 Center for Interdisciplinary AI and Data Science, Ochanomizu University, Tokyo, Japan; 9 School of Advanced Science and Engineering, Waseda University, Tokyo, Japan; 10 Faculty of Biology, Technion-Israel Institute of Technology, Haifa, Israel

**Keywords:** Biophysics, Computational biology and bioinformatics, Biochemistry

## Abstract

Microbial rhodopsins are photoreceptive membrane proteins, which are used as molecular tools in optogenetics. Here, a machine learning (ML)-based experimental design method is introduced for screening rhodopsins that are likely to be red-shifted from representative rhodopsins in the same subfamily. Among 3,022 ion-pumping rhodopsins that were suggested by a protein BLAST search in several protein databases, the ML-based method selected 65 candidate rhodopsins. The wavelengths of 39 of them were able to be experimentally determined by expressing proteins with the *Escherichia coli* system, and 32 (82%, *p* = 7.025 × 10^−5^) actually showed red-shift gains. In addition, four showed red-shift gains >20 nm, and two were found to have desirable ion-transporting properties, indicating that they would be potentially useful in optogenetics. These findings suggest that data-driven ML-based approaches play effective roles in the experimental design of rhodopsin and other photobiological studies. (141/150 words).

## Abstract (web summary)

Inoue, Takeuchi and colleagues propose a machine learning-based protocol to screen rhodopsins for their likelihood to be red-shifted. After experimental verification, their tool shows remarkable success at identifying rhodopsins that showed red-shift gains.

## Introduction

**Fig. 1:**
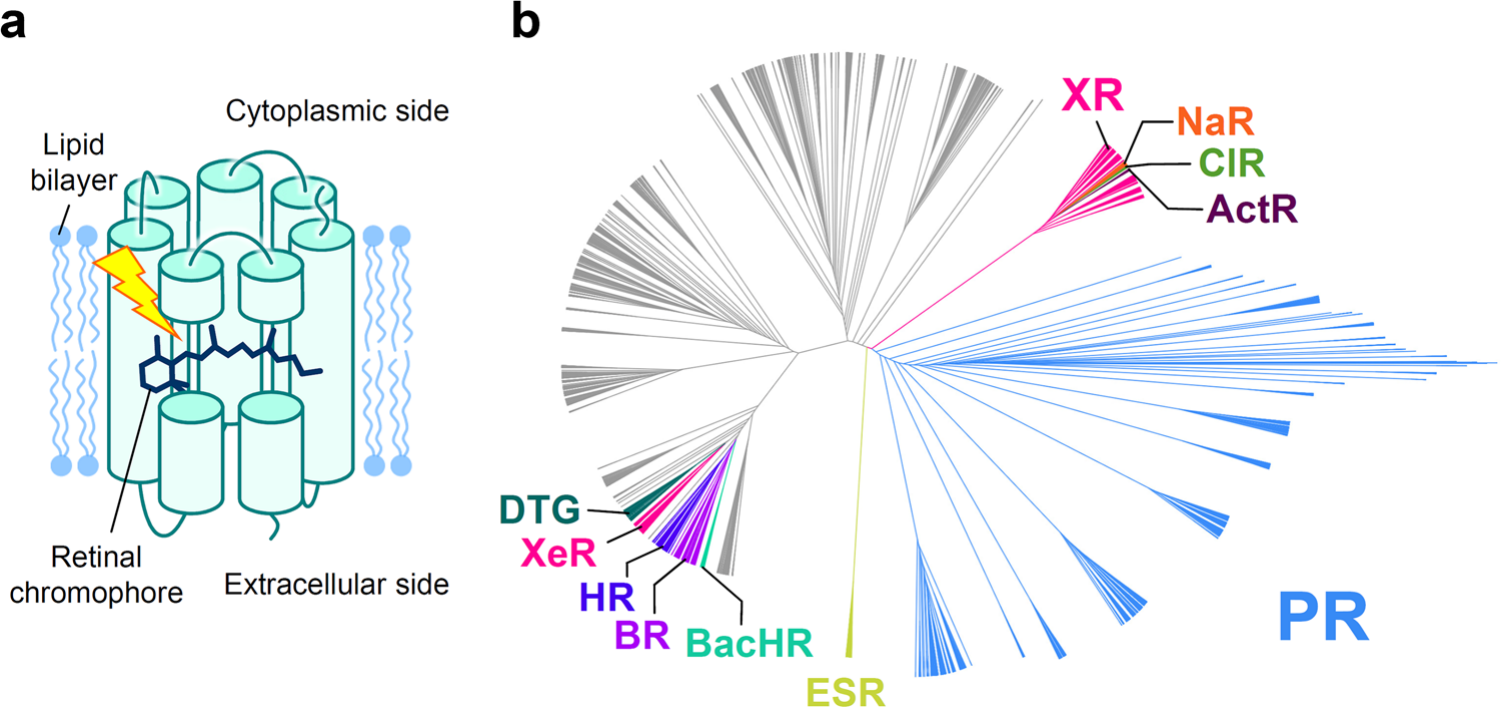
Structure and phylogenetic tree of microbial rhodopsins. **a** Schematic structure of microbial rhodopsins. **b** Phylogenic tree of microbial rhodopsins. The subfamilies of light-driven ion-pump rhodopsins targeted in this study are differently colored; non-ion-pump microbial rhodopsins and ion-pumping microbial rhodopsins from eukaryotic and giant viral origins are shown in gray.

Microbial rhodopsins are photoreceptive membrane proteins widely distributed in bacteria, archaea, unicellular eukaryotes, and giant viruses^1,2^. They consist of seven transmembrane (TM) α helices, with a retinal chromophore bound to a conserved lysine residue in the seventh helix (Fig. 1a). The first microbial rhodopsin, bacteriorhodopsin (BR), was discovered in the plasma membrane of the halophilic archaea *Halobacterium salinarum* (formerly called *H. halobium*)^3^. BR forms a purple-colored patch in the plasma membrane called purple membrane, which outwardly transports H^+^ using sunlight energy^4^. After the discovery of BR, various types of microbial rhodopsins were reported from diverse microorganisms, and recent progress in genome sequencing techniques has uncovered several thousand microbial rhodopsin genes^1,5–7^. These microbial rhodopsins show various types of biological functions upon light absorption, leading to all-*trans*-to-13-*cis* retinal isomerization. Among them, ion transporters, including light-driven ion pumps and light-gated ion channels, are the most ubiquitous (Fig. 1b). Ion-transporting rhodopsins can transport several types of cations and anions, including H^+^, Na^+^, K^+^, halides (Cl^–^, Br^–^, I^–^), NO_3_^–^, and SO_4_^2^,^8–10^. The molecular mechanisms of ion-transporting rhodopsins have been detailed in numerous biophysical, structural, and theoretical studies^1,2^.

In recent years, many ion-transporting rhodopsins have been used as molecular tools in optogenetics to control the activity of animal neurons optically in vivo by heterologous expression^11^, and optogenetics has revealed various new insights regarding the neural network relevant to memory, movement, and emotional behavior^12–15^. However, strong light scattering by biological tissues and the cellular toxicity of shorter wavelength light make precise optical control difficult. To circumvent this difficulty, new molecular optogenetics tools based on red-shifted rhodopsins, which can be controlled by weak scattering and low toxicity longer-wavelength light are urgently needed. Therefore, many approaches to obtain red-shifted rhodopsins have been reported, including gene screening, amino acid mutation based on biophysical and structural insights, and the introduction of retinal analogs^16–18^. The insights obtained in these experimental studies, and further theoretical and computational studies^19–22^ revealed basic physical principle regulating absorption maximum wavelengths (*λ*_max_) of rhodopsins (also called spectral or color-tuning rule) in which the distortion of retinal polyene chain induced by steric interactions with surrounding residues, electrostatic interaction between protonated retinal Schiff base and counterion(s), and polarizability of the retinal binding pocket play essential role^23^. The *λ*_max_ of several rhodopsins could be red-shifted by 20–40 nm without impairing the ion-transport function based on these physicochemical insights^17,24,25^. These are successful examples of knowledge-driven experimental approach. Recently, a new method using a chimeric rhodopsin vector and functional assay was reported to screen the *λ*_max_ and proton transport activities of several microbial rhodopsins that are present in specific environments^26^. This method identified partial sequences of red-shifted yellow (560–570 nm)-absorbing proteorhodopsin (PR), the most abundant outward H^+^-pumping bacterial rhodopsin subfamily, from the marine environment. These works identified several red-shifted rhodopsins^15,16,18,27^. Especially, most successful optogenetic tools are red-shifted channel rhodopsins such as Chrimson^27,28^ and RubyACR^29^ which can induce and inhibit neural firing by absorbing 590 and 610-nm light, respectively. The rational amino acid mutation based on the structural insight further red-shifted the *λ*_max_ of Chrimson to 608 nm^27^. The development of next-generation sequencing technology is expected to continue to more rapidly identify a large number of new rhodopsin genes, including proteins with even longer wavelength-shifted absorption. However, screening of all of them either by experimental or theoretical methods would be very costly. Therefore, a less expensive and more efficient approach to screen red-shifted rhodopsins is needed, and data-driven study is expected as the third class of approach to investigate the color-tuning rule of rhodopsins at low cost.

To estimate the *λ*_max_ of rhodopsins, we recently introduced a data-driven approach^30^. In this previous study, we investigated the statistical relationship between the amino acid types at each position of the seven TM helices and the absorption wavelength of rhodopsins. We constructed a database containing 796 wild-type (WT) rhodopsins and their variants, the *λ*_max_ of which had been reported in earlier studies. Then, we evaluated the strength of the relationship with a data-splitting approach, i.e., the data set was divided into a training set and a test set; the former was used to construct the predictive model, and the latter was used to estimate the predictive ability. The results of this “proof-of-concept’’ study suggested that the *λ*_max_ of an unknown family of rhodopsins could be predicted with an average error of ±7.8 nm, which is comparable to the mean absolute error of *λ*_max_ estimated by the hybrid quantum mechanics/molecular mechanics (QM/MM)^21^ method. Considering the computational cost of both approaches, the data-driven approach was found to be much more efficient than the QM/MM approach, while the latter provides insights on the physical origin controlling *λ*_max_.

Encouraged by this result, in this study, we introduced a machine-learning (ML)-based experimental design method which enables us screening more efficiently the candidates of rhodopsins that are likely to have red-shift gains with data-driven assist compared to the random or knowledge-driven screening. For this aim, we constructed a new dataset of 3022 wild-type putative ion-pump rhodopsins which were collected from public gene databases (NCBI non-redundant protein sequences, and metagenomic proteins^31^ and the *Tara* Oceans microbiome and virome database^32^) and for which *λ*_max_ have not been experimentally investigated yet to explore new red-shifted rhodopsins. The goal of the present study was to identify rhodopsins with *λ*_max_ longer than the wavelengths of the representative rhodopsins in each subfamily of microbial rhodopsins for which the *λ*_max_ has already been reported (base wavelengths). Here, we call the degrees of red-shift of the wavelength from the base wavelength the “red-shift gain”. We focus on rhodopsins with large red-shift gains because this would lead to the identification of amino acid types and residue positions that play important roles in red-shifting absorption wavelengths. Also, it is practically important in optogenetics applications to have a wide variety of ion-pumping rhodopsins from each subfamily to construct a new basis for rhodopsin toolboxes with red-shifted absorption and various types of ion species that can be transported. We constructed the ML-based experimental design method so that it could properly predict the expected red-shift gains, and applied this new method to 3022 putative ion-pumping rhodopsins derived from archaeal and bacterial origins that can be easily expressed in *Escherichia coli* (Fig. 1b).

We conducted experiments by introducing the synthesized rhodopsin genes into *E. coli* to measure the absorption wavelengths of 65 candidates for which the ML-based experimental design method predicted that the expected gains were >10 nm. Of these 65 selected candidates, 39 showed substantial coloring in *E. coli* cells, 32 showed actual red-shift gains, 6 showed blue-shifts, and 1 showed no change, i.e., 82% (=32/39, 7.025 × 10^−5^) of the selected candidates showed actual red-shift gains. We then investigated the ion-transportation properties of the rhodopsins for which the red-shift gains were >20 nm, and found that some actually had desirable ion-transporting properties, suggesting that they (and their variants) could potentially be used as new optogenetics tools. Furthermore, the differences in the amino acid sequences of the newly examined rhodopsins and the representative ones in the same subfamily could be used for further investigation of the red-shifting mechanisms. This result suggests that it should be possible to find rhodopsins that have desired properties without conducting exhaustive biological experiments, and suggests that data-driven ML-based approaches should play effective roles in the experimental design of rhodopsin and other photobiological studies.

## Results

### Construction of an ML-based experimental design method for predicting expected red-shift gain

**Fig. 2:**
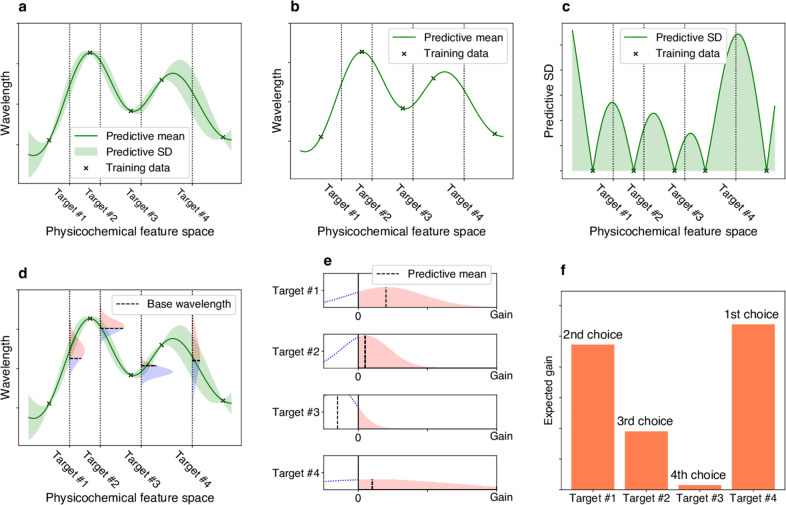
Illustrations of exploration–exploitation for screening rhodopsins with red-shift gain. **a** Bayesian prediction model constructed using the current training data (black crosses). The prediction model is represented by the predictive mean and predictive standard deviation (SD). The horizontal axis schematically illustrates the space of proteins defined through physicochemical features. The four vertical dotted lines indicate target proteins (candidates to synthesize). **b** Predictive mean. This function is defined as the expected value of the probabilistic prediction by the Bayesian model. **c** Predictive SD. Since the predictive SD represents the uncertainty of the prediction, it has a larger value when the training data points do not exist nearby. **d** The distributions on the vertical dotted lines represent the predictive distributions, and the horizontal dashed lines are the base wavelengths of the target points. The base wavelength is different for each target point because it depends on the subfamily of the protein. **e** The density of the predictive distribution of each target protein on its red-shift gain value. The gain is defined as the predicted wavelength subtracted by the base wavelength, and if it is negative, the value is truncated as 0. This can be seen as a “benefit” that can be obtained by observing the target protein. **f** Expected value of the red-shift gain. This provides a ranking list from which the next candidates to be experimentally investigated can be determined. Target #4 has the largest expected gain, although target #1 has the largest increase in the predictive mean compared with base wavelength in **e**. Because of its larger SD (as shown in **a**, **c**, **d**, and **e**), target #4 is probabilistically expected to have a larger gain than the other targets.

To screen rhodopsins that would have large red-shift gains, it is necessary to consider the uncertainty of prediction in the form of “predictive distributions”^33^. By using predictive distributions, it is possible to consider appropriately the “exploration–exploitation trade-off” in screening processes^34,35^, where exploration indicates an approach that prefers candidates with larger predictive variances, and exploitation indicates an approach that prefers candidates with longer predictive mean wavelengths (Fig. 2). Here, the term “exploration–exploitation’’ is a technical term used in the fields of active learning and experimental design, and “explorations*’’* in the title of this paper is used in a broader sense and is not directly related to the former technical terminology. We employed a Bayesian modeling framework to compute the predictive distributions of candidate rhodopsin red-shift gains. We then consider an exploration–exploitation trade-off by selecting candidate rhodopsins based on a criterion called “expected red-shift gains”.

**Fig. 3:**
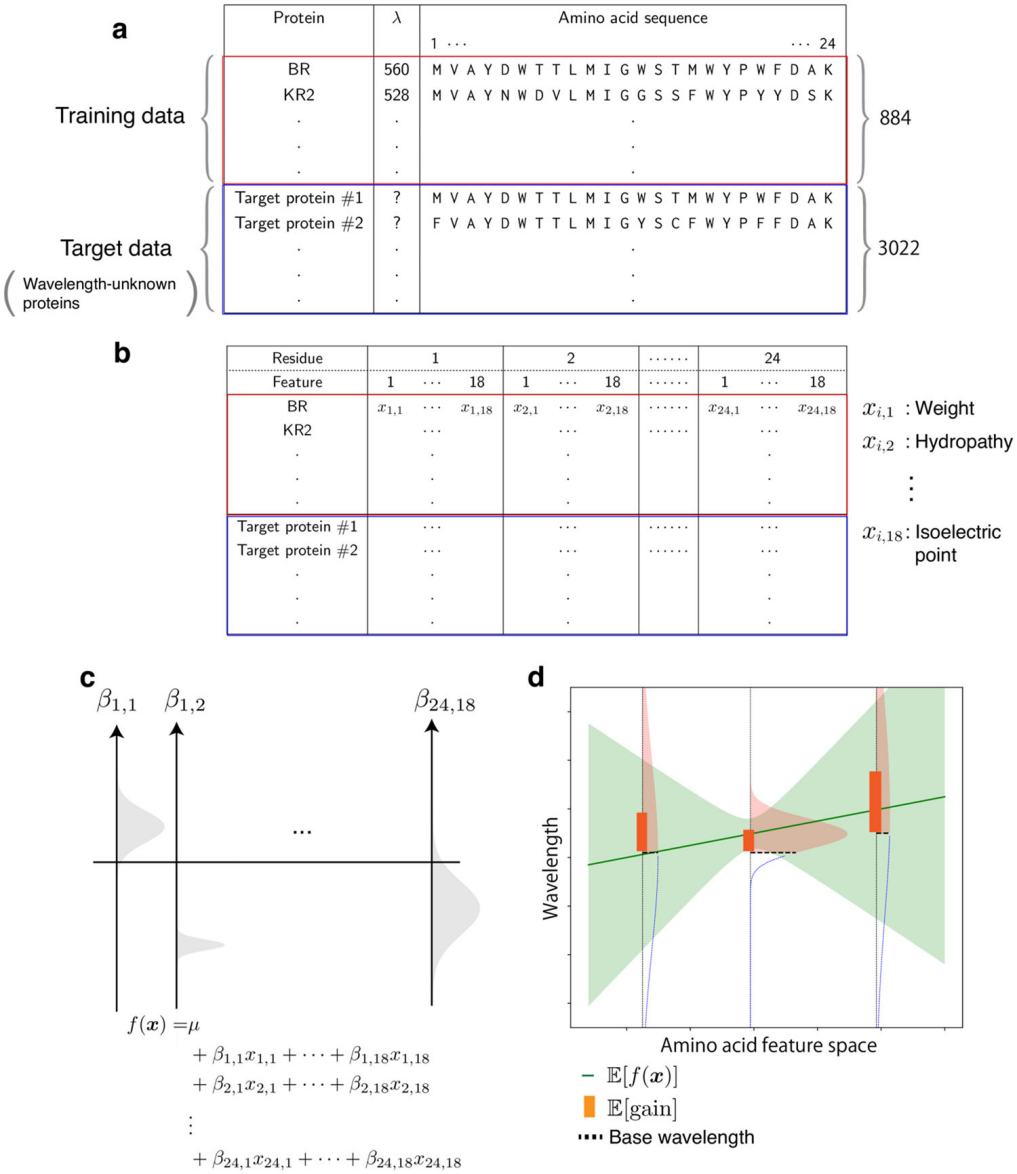
Overview of the ML-based exploration of natural red-shifted rhodopsins. **a** Using existing experimental data, a training data set consisting of pairs of a wavelength *λ*_max_ and an amino acid sequence was constructed. A particular focus was placed on the 24 amino acid residues around the retinal chromophore to build an ML-based prediction model. A set of protein sequences with no known wavelength was also collected as target proteins. **b** All amino acid sequences were transformed into physicochemical features, leading to 24 ×18 = 432 dimensional numerical representations of each protein. **c** A linear regression model was constructed using the Bayesian approach. Each regression coefficient *β*_*i*,*j*_ was estimated as a distribution (shown as a gray region). The broadness of these distributions represent the uncertainty of the current estimation. **d** The expected red-shift gain values were evaluated for the target proteins. The green region is the standard deviation of the prediction. The red shaded region in the vertical distribution corresponds to the probability that the wavelength is larger than the base wavelength (dashed line), which is determined by the subfamily of the microbial rhodopsin. The bar represents the expected red-shift gain, defined by the expected value of the increase from the base wavelength.

To compute the expected red-shift gains of a wide variety of rhodopsins, we developed ML-based experimental design method based on the statistical analysis in our previous study^30^. Figure 3 shows a schematic illustration of the ML-based experimental design method. First, we added 88 WT microbial rhodopsins and their variants for which the *λ*_max_ had recently been reported in the literature or determined by our experiments, to a previously reported data set^30^. In other words, the new training data set consisted of the amino acid sequences and *λ*_max_ of 884 WT microbial rhodopsins and their variants (Supplementary Data 1). Second, the new ML model used only *N* = 24 residues located around the retinal chromophore (Supplementary Fig. 1) because our previous study^30^ indicated that amino acid residues at these 24 positions play significant roles in predicting absorption wavelengths (Fig. 3a). Third, *M* = 18 amino acid physicochemical features (Supplementary Data 2) were used as inputs in the ML model, as opposed to the amino acid types used in the previous statistical analysis. This enabled us to predict the absorption wavelengths of a wide range of target rhodopsins that contain unexplored amino acid types in the training data at certain positions. Therefore, an amino acid sequence is transformed into an *M* × *N* = 432 dimensional feature vector ${\boldsymbol{x}} \in {\Bbb R}^{MN}$ by concatenating *x*_*i,j*_, the *j*-th feature of the *i*-th residue (Fig. 3b). We consider a linear prediction model $f\left( {\boldsymbol{x}} \right) = \mu + \mathop {\sum}\nolimits_{i = 1}^N {\mathop {\sum}\nolimits_{j = 1}^M {\beta _{i,j}x_{i,j}} }$, where *β*_*i,j*_ is the parameter for the *j*-th feature of the *i*-th residue, and *μ* is the intercept term.

Finally, to consider the exploration–exploitation trade-off appropriately in the screening process, we introduce a Bayesian modeling framework, which allows us to compute the predictive distributions of red-shift gains. Specifically, we employed Bayesian sparse modeling called BLASSO^36^ (see the Methods section for details). This enables us to provide not only the mean, but also the variance of the predicted wavelengths. Unlike classical regression analysis, BLASSO regards the model parameters *β*_*i,j*_ and *μ* as random variables generated from underlying distributions, as illustrated in Fig. 3c. Therefore, the wavelength prediction *f*(***x***) is also represented as a distribution. The red-shift gain is defined as gain = max(*f*(***x***)−*λ*_base’_0), where *λ*_base_ is the wavelength of the representative rhodopsin in the same subfamily whose *λ*_max_ has been experimentally determined and reported in the literature (Supplementary Data 3). Note that the red-shift gain is positive if *f* (***x***) is greater than *λ*_base_; otherwise, it takes the value of zero. Since *f* (***x***) is regarded as a random variable in BLASSO, the red-shift gain is also regarded as a random variable. Therefore, we employ the expected value of the red-shift gain, denoted by ${\Bbb E}[{\mathrm{gain}}]$, as the screening criterion where ${\Bbb E}$ represents the expectation of a random variable. Illustrative examples of ${\Bbb E}[{\mathrm{gain}}]$ are shown in Fig. 3d. Unlike the simple expectation of the wavelength prediction ${\Bbb E}[f({\boldsymbol{x}})]$, ${\Bbb E}[{\mathrm{gain}}]$ depends on the variance of the predictive distribution (For example, ${\Bbb E}[{\mathrm{gain}}]$ of target #4 is larger than #1 in Fig. 2f though ${\Bbb E}\left[ {f\left( {\boldsymbol{x}} \right)} \right] - \lambda _{{\mathrm{base}}}$ of #4 is smaller than #1 in Fig. 2e). This encourages the exploration of rhodopsin candidates having large uncertainty (for exploration), as opposed to only those having longer wavelengths with high confidence (for exploitation).

### Screening potential red-shifted microbial rhodopsins based on expected red-shift gains

The target data set to explore red-shifted microbial rhodopsins was constructed with putative microbial rhodopsin genes collected by a protein BLAST (blastp) search^37^ of the NCBI non-redundant protein and metagenome databases^31^, as well as the *Tara* Oceans microbiome and virome databases^32^. As a result, we obtained a non-redundant data set of 5558 microbial rhodopsin genes (Fig. 1b). The sequences were aligned by ClustalW and categorized to subfamilies of microbial rhodopsins based on the phylogenic distances, as reported previously^38^. Among these, 3022 rhodopsin genes, which did not have identical sequences in the training data and from bacterial and archaeal origins, were extracted because their *λ*_max_ can be easily measured by expressing in *E. coli* cells. We calculated the ${\Bbb E}[{\mathrm{gain}}]$ of these 3022 genes (Supplementary Data 4), and then selected 65 genes of putative light-driven ion-pump rhodopsins showing an ${\Bbb E}[{\mathrm{gain}}]$ >10 nm for further experimental evaluation, as ion-pump rhodopsins can be used as new optogenetics tools.

### Experimental measurement of the absorption wavelengths of microbial rhodopsins showing high red-shift gains

**Fig. 4:**
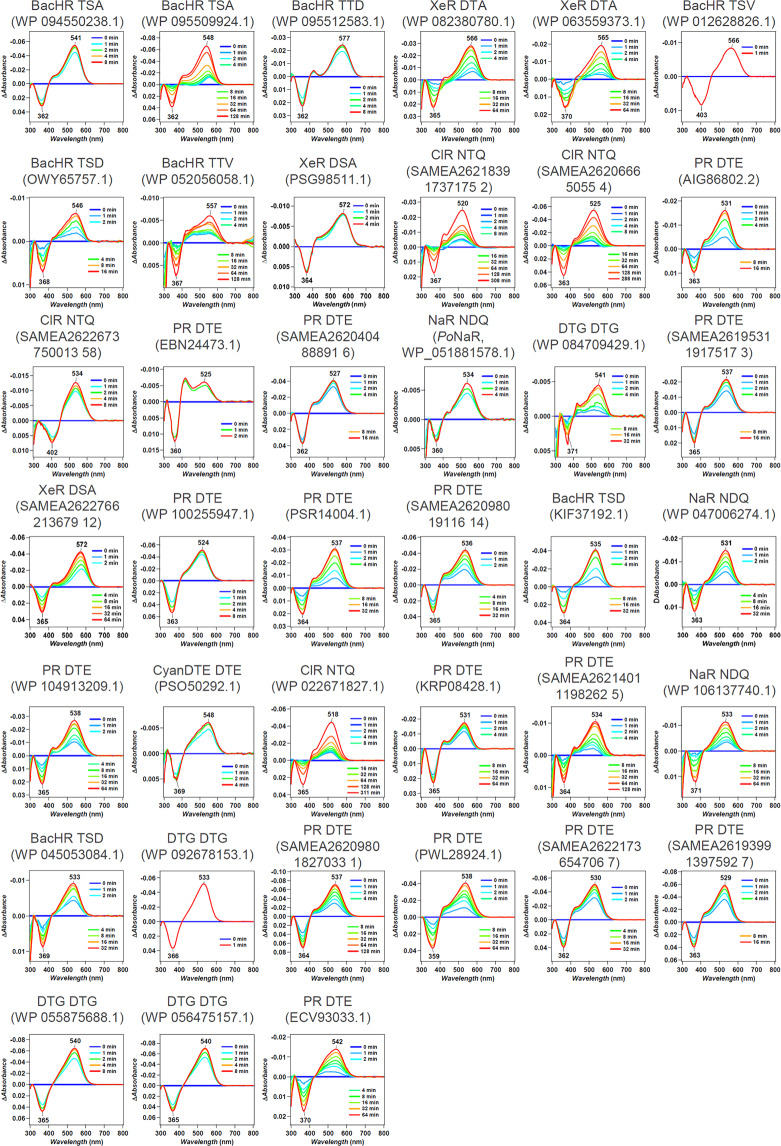
*λ*_max_ of 39 microbial rhodopsins in solubilized *E. coli* membrane observed upon hydroxylamine bleach reaction. The difference absorption spectra between before and after hydroxylamine bleaching reaction of microbial rhodopsins in solubilized *E. coli* membrane. The *λ*_max_ of each rhodopsin was determined by the peak positions of the absorption spectra of the original proteins, and the absorption of retinal oxime produced by the reaction of retinal Schiff base and hydroxylamine was observed as a negative peak at ~360–370 nm.

**Fig. 5:**
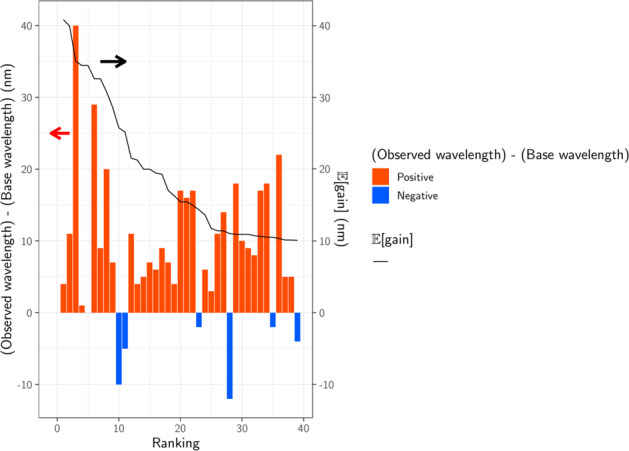
Observed wavelengths and expected red-shift gains. The predicted and observed red-shift (and blue-shift) gains for the 39 candidate rhodopsins that showed substantial coloring in *E. coli* cells. Differences between observed and base wavelengths are shown by the bars. The red bars indicate red-shift from the base wavelength, while the blue bars indicate observed wavelengths that were shorter than the base wavelengths. Proteins are sorted in the descending order by ${\Bbb E}[{\mathrm{gain}}]$, as shown by the black line. Among the 39 candidates, 32 (82%) showed red-shift gains, suggesting that the proposed ML-based model can screen red-shifted rhodopsins more efficiently than random choice.

We synthesized the selected 65 genes that showed an ${\Bbb E}[{\mathrm{gain}}]$ > 10 nm. These were then introduced into *E. coli* cells, and the proteins expressed in the presence of 10 μM all-*trans* retinal. As a result, 39 *E. coli* cells showed substantial coloring, indicating high expression of folded protein, and their *λ*_max_ were determined by observing ultraviolet (UV)-visible absorption changes upon bleaching of the expressed rhodopsins through a hydrolysis reaction of their retinal with hydroxylamine, as previously reported^30^ (Fig. 4). The observed gains were compared with the ${\Bbb E}[{\mathrm{gain}}]$ shown in Table 1. A full list of unexpressed genes is shown in Supplementary Data 5. In total, 32 out of 39 genes showed a longer wavelength than their base wavelength (that is, positive red-shift gain; Fig. 5), suggesting that our ML-based model can significantly improve the efficiency of screening to explore new red-shifted microbial rhodopsins compared with random sampling (*p* = 7.025 × 10^−5^ by a binomial test assuming that the probability of red-shift gain for random choice is 50%).

**Table 1:** Predicted and observed gains of 39 microbial rhodopsins expressed in *E. coli*.

Origin	Accession	Subfamily	Motif	Base wavelength/nm	${\Bbb E}$ [gain]	Observed wavelength/nm	(Observed wavelength)–(base wavelength)/nm
*Rubricoccus marinus*	WP 094550238.1	BacHR	TSA	537	40.7	541	4
*Rubrivirga marina*	WP 095509924.1	BacHR	TSA	537	39.8	548	11
*Rubrivirga marina*	WP 095512583.1	BacHR	TTD	537	35.5	577	40
*Bacillus* sp. CHD6a	WP 082380780.1	XeR	DTA	565	35.3	566	1
*Bacillus horikoshii*	WP 063559373.1	XeR	DTA	565	35.3	565	0
*Cyanothece* sp. PCC 7425	WP 012628826.1	BacHR	TSV	537	32.9	566	29
*Cyanobacterium* TDX16	OWY65757.1	BacHR	TSD	537	32.9	546	9
*Myxosarcina* sp. GI1	WP 052056058.1	BacHR	TTV	537	31.2	557	20
*Nanohaloarchaea* archaeon SW 7 43 1	PSG98511.1	XeR	DSA	565	29.2	572	7
Metagenome sequence	SAMEA2621839 1737175 2	ClR	NTQ	530	25.7	520	−10
Metagenome sequence	SAMEA2620666 5055 4	ClR	NTQ	530	25.1	525	−5
*Nonlabens* sp. YIK11	AIG86802.2	PR	DTE	520	21.5	531	11
Metagenome sequence	SAMEA2622673 750013 58	ClR	NTQ	530	21.4	534	4
Metagenome sequence	EBN24473.1	PR	DTE	520	20.0	525	5
Metagenome sequence	SAMEA2620404 88891 6	PR	DTE	520	20.0	527	7
*Parvularcula oceani*	WP_051881578.1	NaR	NDQ	525	19.7	534	9
*Rubrobacter* aplysinae	WP 084709429.1	DTG	DTG	535	19.5	541	6
Metagenome sequence	SAMEA2619531 1917517 3	PR	DTE	520	18.0	537	17
Metagenome sequence	SAMEA2622766 213679 12	XeR	DSA	565	17.8	572	7
*Reinekea forsetii*	WP 100255947.1	PR	DTE	520	17.1	524	4
*Bacteroidetes bacterium*	PSR14004.1	PR	DTE	520	15.4	537	17
*Metagenome sequence*	SAMEA2620980 19116 14	PR	DTE	520	15.4	536	16
*Hassallia byssoidea* VB512170	KIF37192.1	BacHR	TSD	537	15.1	535	−2
*Erythrobacter gangjinensis*	WP 047006274.1	NaR	NDQ	525	13.7	531	6
*Pontimonas salivibrio*	WP 104913209.1	PR	DTE	520	12.2	538	18
*Cyanobacteria* bacterium QH 1 48 107	PSO50292.1	CyanDTE	DTD	545	12.0	548	3
*Sphingopyxis baekryungensis*	WP 022671827.1	ClR	NTQ	530	11.0	518	−12
*Sphingobacteriales* bacterium BACL12 MAG120802bin5	KRP08428.1	PR	DTE	520	10.9	531	11
Metagenome sequence	SAMEA2621401 1198262 5	PR	DTE	520	10.9	534	14
*Spirosoma oryzae*	WP 106137740.1	NaR	NDQ	525	10.8	533	8
*Aliterella atlantica*	WP 045053084.1	BacHR	TSD	537	10.8	533	−4
*Rosenbergiella nectarea*	WP 092678153.1	DTG	DTG	535	10.8	533	−2
Metagenome sequence	SAMEA2620980 1827033 1	PR	DTE	520	10.4	537	17
*Fluviicola* sp. XM24bin1	PWL28924.1	PR	DTE	520	10.4	538	18
Metagenome sequence	SAMEA2622173 654706 7	PR	DTE	520	10.4	530	10
Metagenome sequence	SAMEA2619399 1397592 7	PR	DTE	520	10.4	529	9
*Sphingomonas* sp. Leaf34	WP 055875688.1	DTG	DTG	535	10.3	540	5
*Sphingomonas* sp. Leaf38	WP 056475157.1	DTG	DTG	535	10.3	540	5
Metagenome sequence	ECV93033.1	PR	DTE	520	10.3	542	22

### Ion-transport function of red-shifted microbial rhodopsins

**Fig. 6:**
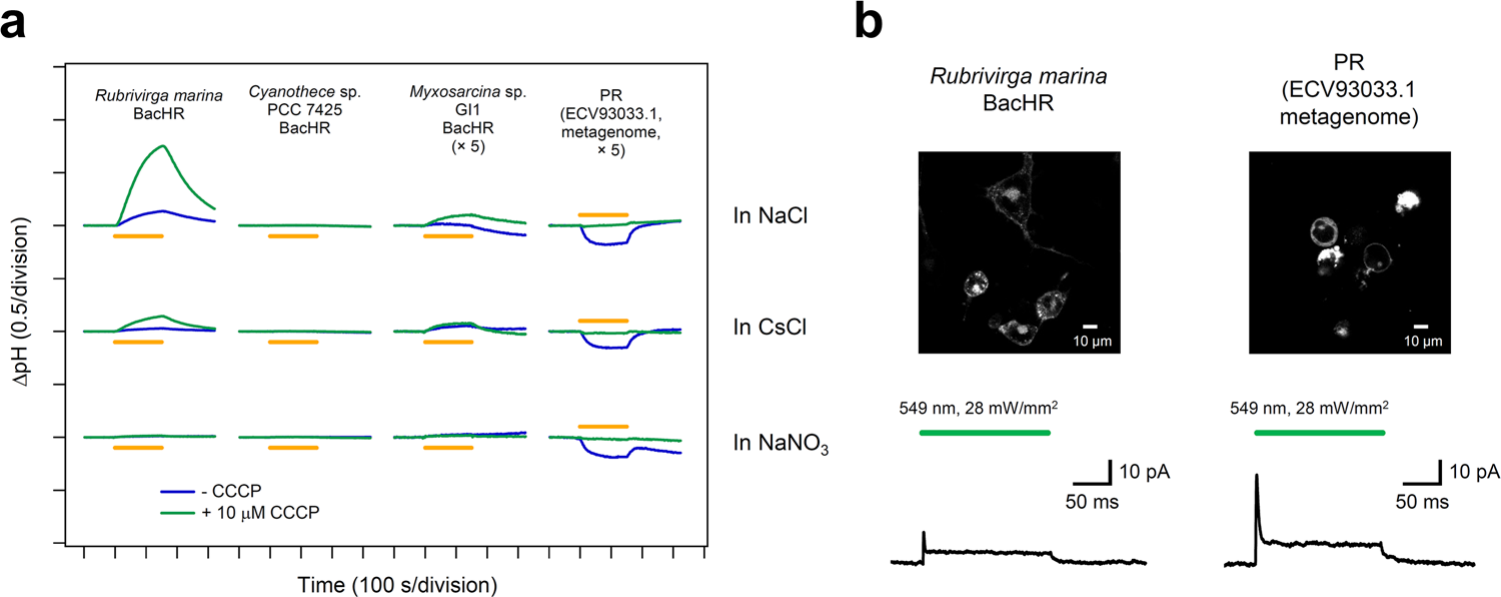
Light-driven ion-transport activities of microbial rhodopsins showed longer *λ*_max_. **a** The light-induced pH change in the external solvent of *E. coli* cells expressing four microbial rhodopsins that showed a *λ*_max_  ≥ 20 nm longer than the base wavelength of the subfamily. The data obtained without and with 10 μM CCCP are indicated by the blue and green lines, respectively, in 100 mM NaCl, CsCl, and NaNO_3_. Light was illuminated for 150 s (yellow solid lines). **b** *Rubrivirga marina* BacHR or PR (ECV93033.1 metagenome) were expressed in the membrane of ND7/23 cells (top image) and generated positive photocurrent in response to a green light pulse (200 ms, 549 nm, 28 mW/mm^2^). The traces in the bottom are typical records at a holding potential of 0  mV.

Overall, 4 of the 39 rhodopsins showed red-shifted absorption ≥20 nm compared with the base wavelengths (Table 1): three were halorhodopsins (HRs) from bacterial species^10,39,40^ (to distinguish classical HRs from archaeal species, these are hereafter referred to as bacterial-halorhodopsins [BacHRs]), and one was a PR^41^. Their ion-transport activities were then investigated by expressing in *E. coli* cells and observing the pH change in external solvent whose pH was initially set to 7 (Fig. 6a). Upon light illumination, BacHRs from *Rubrivirga marina* and *Myxosarcina* sp. GI1 showed alkalization of external solvent, which was enhanced by addition of the protonophore (CCCP), which increases the H^+^ permeability of the cell membrane, and the light-dependent alkalizations disappeared when anions were exchanged from Cl^–^ to NO_3_^–^, indicating that these were light-driven Cl^–^ pumps, similar to other rhodopsins in the same BacHR subfamily^10,39^. By contrast, *Cyanothece* sp. PCC 7425 did not show any substantial transport. While no transporting function can be attributed to the heterologous expression in *E. coli*, it would have considerably different molecular properties from other BacHRs. PR from a metagenome sequence (ECV93033.1) showed acidification of external solvent that was abolished by the addition of CCCP and was independent from ionic species in the solvent. Hence, this was a new red-shifted outward H^+^ pump compared with typical PRs whose *λ*_max_ are present at ca. 520 nm^41^. Furthermore, these rhodopsins are needed to be functional in mammalian cells for their optogenetic applications. To verify this issue, we carried out electrophysiological experiment to measure the photocurrent of BacHRs from *Rubrivirga marina* and PR from a metagenome sequence (ECV93033.1) in mammalian cells (ND7/23; Fig. 6b). Both of them showed substantial photocurrent even in the mammalian cells. These light-driven ion-pumping rhodopsins with red-shifted *λ*_max_ have the potential to be applied as new optogenetics tools, and thus, warrant further study in the near future.

## Discussion

Microbial rhodopsins show a wide variety of *λ*_max_ by changing steric and electrostatic interactions between all-*trans* retinal chromophores and surrounding amino acid residues. An understanding of the color-tuning rule enables more efficient screening and the design of new red-shifted rhodopsins that have value as optogenetics tools, and our ML-based data-driven approach therefore provides a new basis to identify color-regulating factors without assumptions.

We previously demonstrated that an ML-based model based on ∼800 experimental results could predict the *λ*_max_ of microbial rhodopsins with an average error of ±7.8 nm. Encouraged by this result, in the present study, we constructed a new ML-based model to compute expected red-shift gains for a wide range of unknown families of microbial rhodopsins. As a result, 32 out of 39 microbial rhodopsins were found to have red-shifted absorption compared with the base wavelengths of each subfamily of microbial rhodopsins (Table 1), suggesting that our data-driven ML approach can screen red-shifted microbial rhodopsin genes more efficiently than random choice (*p* = 7.025 × 10^−5^).

**Fig. 7:**
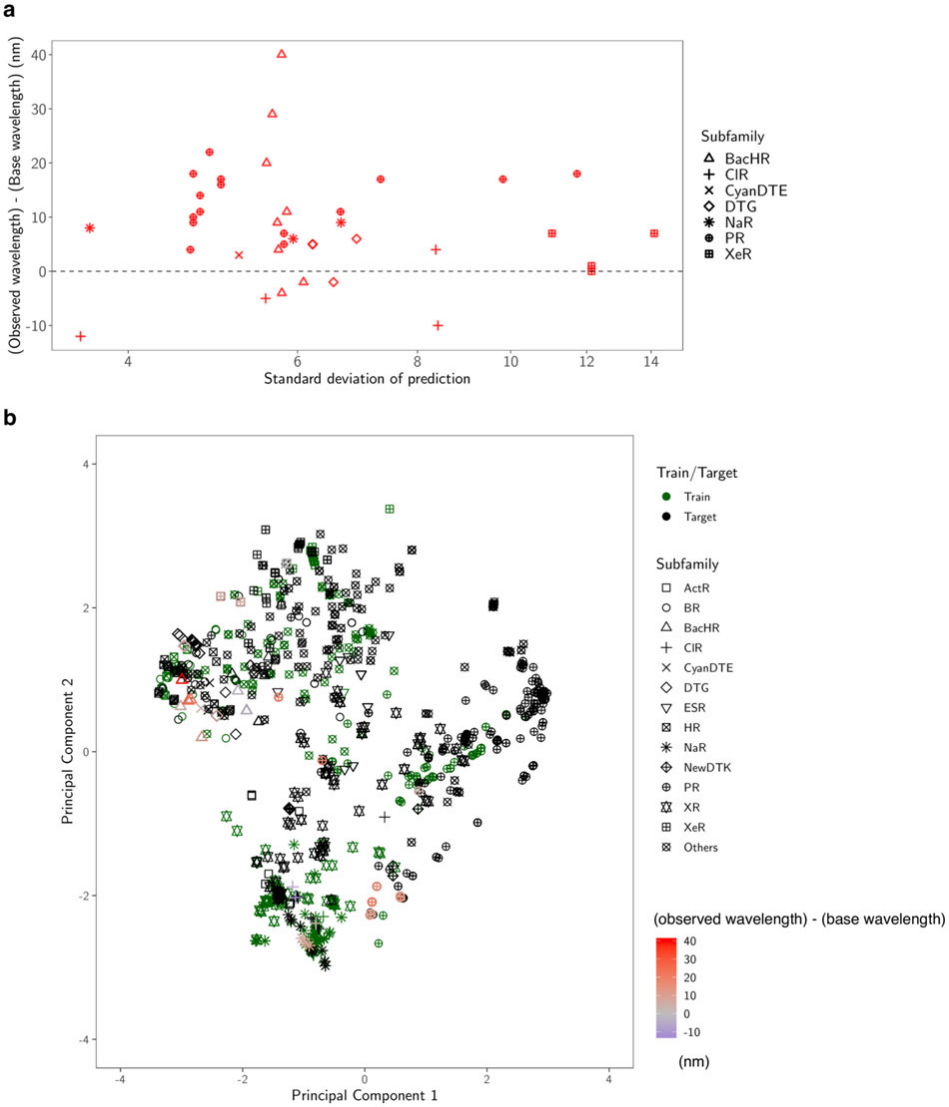
Diversity of the selected proteins. **a** Predicted standard deviation (horizontal axis) vs. observed gain (vertical axis). The marker shape represents the subfamily of each protein. **b** Two-dimensional projection created by principal component analysis. The original *d* = 432 dimensional feature space is projected onto the first two principal component directions. The first component (horizontal axis) explains 33% of the total variance of the original space, and the second (vertical axis) explains 17%. The green markers are the training data, and the black markers are the target data. For the synthesized proteins, differences in the observed and base wavelengths are shown by the color map. The results indicate that, by considering the exploration–exploitation trade-off, it was possible to make a red-shift protein screening process that considered not only the expected value of the prediction, but also the uncertainty.

**Fig. 8:**
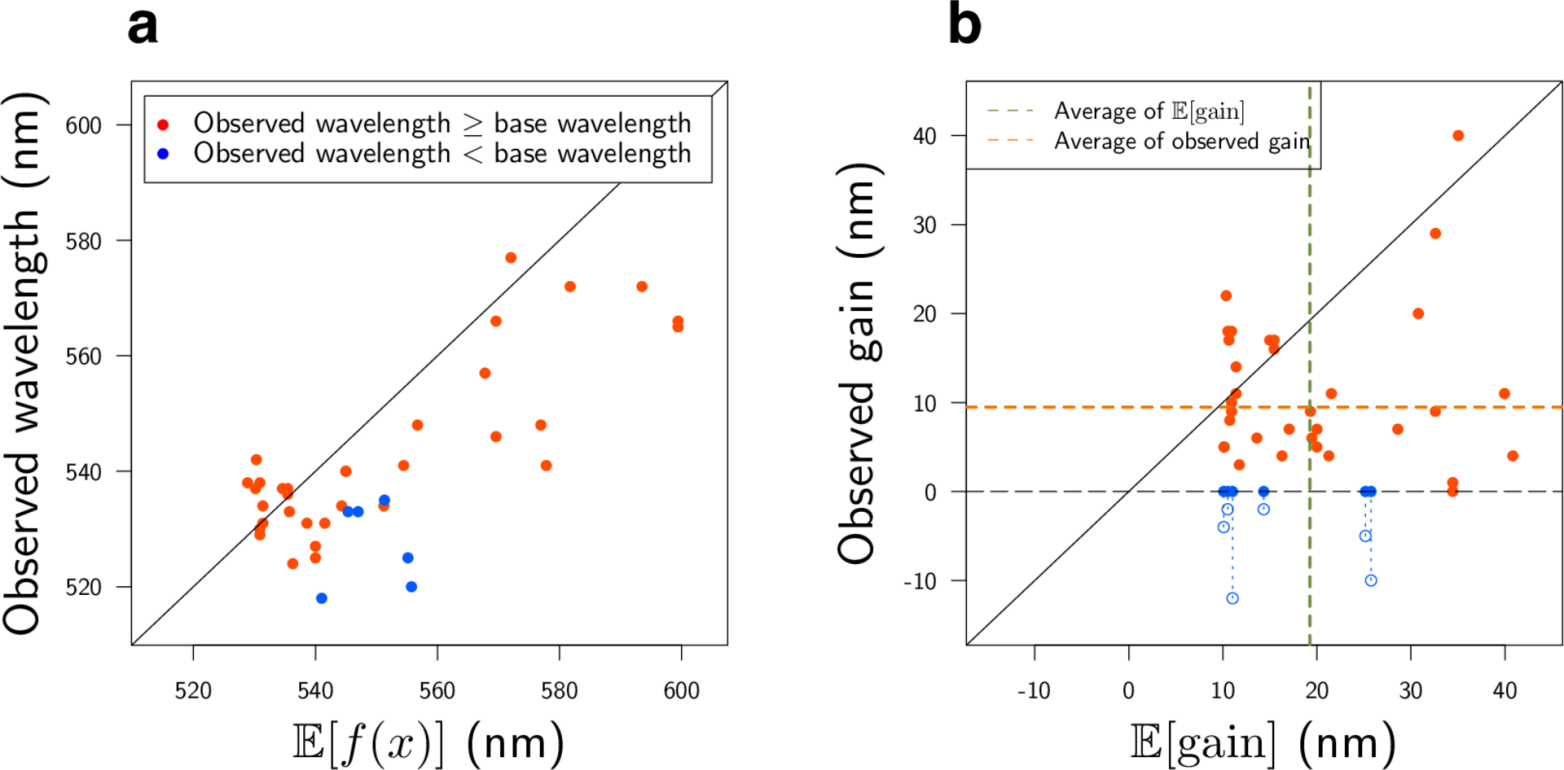
Comparisons of experimental observations and ML predictions. In these two plots, the red points have longer observed wavelengths than the base wavelength *λ*_base_, while the blue points have shorter observed wavelengths than *λ*_base_. **a** ML-based prediction of *λ*_max_ (horizontal axis) vs. experimentally observed *λ*_max_ (vertical axis). **b** Expected red-shift gain (horizontal axis) vs. observed gain (vertical axis). Since we selected rhodopsins having expected red-shift gains of ≥10 nm, all the points on the horizontal axis are ≥10 nm. The observed gain, defined by max (*λ*_max_−*λ*_base_,0), is nonnegative by definition. For blue points whose observed gain is equal to 0, the value of *λ*_max_−*λ*_base_ is also shown as blue outlined circles. The green and orange dashed lines are the averages of the horizontal and vertical axes (19.2 nm and 9.5 nm), respectively. The results indicate that the observed wavelengths and red-shift gains tended to be smaller than the predicted ones. We conjecture that these differences between the observed and predicted wavelengths are due to modeling errors (see the Discussion for details).

By considering the exploration–exploitation trade-off, that is, to consider not only the expected value of the prediction, but also the uncertainty, it was possible to construct a red-shift protein screening process, as shown in Fig. 7. Figure 7a shows the relationships between the prediction uncertainty (as measured by the standard deviation) and the observed red-shift gains. It can be seen that rhodopsins with red-shift gain are found in areas of not only low (small standard deviation), but also high prediction uncertainty (large standard deviation). Figure 7b shows the two-dimensional projection of the *d* = 432 dimensional feature space by principal component analysis. It can be seen that red-shift gains (red) are found for target proteins not only close to training proteins (green), but also far from training proteins. Figure 8 shows that the observed wavelengths and red-shift gains tend to be smaller than the predicted ones. We conjecture that these differences between the observed and predicted wavelengths and red-shift gains are due to modeling errors, possibly caused by a lack of sufficient information (e.g., three-dimensional structures) and modeling flexibility (e.g., nonlinear effects); in other words, rhodopsins having high prediction values partly by modeling errors have a high chance of being selected. Therefore, it would be valuable to develop a statistical methodology to eliminate selection bias due to modeling errors.

Four rhodopsins showed red-shifted absorption ≥20 nm than the base wavelength, three of which showed light-driven ion-transport function. Interestingly, while one BacHR from *Rubrivirga marina* (accession No.: WP 095512583.1) showed a 40-nm longer *λ*_max_ (577 nm) than the base wavelength, another 11-nm red-shifted BacHR (WP 095509924.1) was also identified from the same bacteria (Table 1). These BacHRs are highly similar to each other (55.2% identity and 70.6% similarity), and only four of 24 amino acid residues around the retinal chromophore differ. Hence, *R. marina* evolved two BacHRs with 29-nm different *λ*_max_ by a small number of amino acid replacements; the amino acid residue(s) responsible for this color-tuning should be investigated in the future.

The differences in amino acids in three of 24 retinal-surrounding residues are known to play a color-tuning role in natural rhodopsins without affecting their biological function. These correspond to positions 93, 186, and 215 in BR (BR Leu93, Pro186, and Ala215, respectively)^17^. Position 93 is known to be diversified in the PR family (the well-known position 105 in PRs). Green-light-absorbing PRs (GPRs) have leucine as a BR, whereas glutamine is conserved in blue-light-absorbing PRs^5,26^. This color-tuning effect by the difference between leucine and glutamine is known as the “L/Q-switch”^42^. Interestingly, while 29.8% of 3022 candidate genes have glutamine at this position, all 39 genes whose large red-shift gains were suggested by our ML-based model have amino acids other than glutamine, which suggests that our ML-based model avoided the genes having glutamine at position 93. Especially, 12 (37.5%) of 32 genes that actually showed red-shifted absorption compared with the base wavelengths had methionine at this position (Supplementary Data 6), which is substantially higher than the proportion of methionine-conserving genes in the 3022 candidates (16.1%). The red-shifting effect of the L-to-M mutation of this residue in GPRs previously reported^42^ and the current result imply that many rhodopsins have evolved methionine to absorb light with longer wavelengths. Position 215 in BR is also known to have a color-tuning role. The mutation from alanine to threonine or serine (A/TS switch) has a blue-shifting effect of 9–20 nm^17,43–45^. Five of six genes that showed blue-shifted *λ*_max_ compared with the base wavelengths have threonine or serine at this position, suggesting that these types of genes should be avoided to explore red-shifted rhodopsins. By contrast, asparagine was conserved in more than half (58.4%) of the 3022 candidate genes, especially in those belonging to the PR subfamily. A substantial portion (37.5%) of the genes with red-shifted absorption compared with the base wavelengths also had asparagine at this position (Supplementary Data 6). The A-to-N mutation at this position had a smaller effect (4–7 nm)^30,44^ than that of the A-to-S/T mutation; thus, the difference between alanine and asparagine is not so critical to explore red-shifted rhodopsins. Position 186 in BR is proline in most microbial rhodopsins (in 98.7% of the 3022 candidate genes), and the mutation to non-proline amino acids induces red-shift of absorption^17^. We identified sodium pump rhodopsin (NaR) from *Parvularcula oceani*, which also has a threonine at this position, and showed 10-nm longer absorption than the base wavelength. Although genes having non-proline amino acids are rare in nature, it would be beneficial to identify new red-shifted rhodopsins. These results indicate that ML-based modeling can provide insights for identifying new functional tuning rules for proteins based on specific amino acid residues.

The number of reported microbial rhodopsin genes is rapidly increasing due to the development of next-generation sequencing techniques and microbe culturing methods. New microbial rhodopsins with molecular characteristics suitable for optogenetics applications are expected to be included in upcoming genomic data. Data-driven approaches would be able to efficiently suggest promising rhodopsins which should be investigated preferentially. Although the absorption of the most red-shifted rhodopsin found in this study (BacHR from *Rubrivirga marina*, *λ*_max_ = 577 nm) is shorter than the peak activation wavelength of e*Np*HR3.0 (590 nm) which is extensively used in optogenetic studies^46^, our ML-based model could be expected to reduce the costs associated with identifying red-shifted rhodopsins from upcoming genomic data. Especially, we expect that our ML-based model could be applied to ion channel and enzymatic rhodopsins, which were not a focus of this study because of their eukaryotic origins; however, their use in optogenetics research could help identify more useful optogenetics tools with red-shifted absorption in the future.

## Methods

### Experimental design

The objective of this study was to introduce and demonstrate the effectiveness of a data-driven experimental design method to screen candidates for rhodopsin proteins with desired properties from more than several thousand candidates identified in various microbial species. To this end, we constructed a training dataset for developing a ML model and a target dataset for screening targets (Construction of training and target data sets). A machine learning model was constructed using the training dataset (ML modeling), which was used to select the 65 candidates from 3022 in the target dataset. The protein expressions of selected candidates were induced (Protein expression), and the absorption spectra and *λ*_max_ of the selected rhodopsins were measured (Measurement of the absorption spectra and *λ*_max_ of rhodopsins by bleaching with hydroxylamine). Furthermore, we investigated the ion-transportation properties of the rhodopsins that showed large red-shift gains (Ion-transport assay of rhodopsins in *E. coli* cells). Statistical significance of the effectiveness of the data-driven experimental design method was assessed by a binomial test.

### Construction of training and target data sets

In this study, we constructed a new training data set (Supplementary Data 1) by adding 88 genes for which the *λ*_max_ had recently been reported in the literature or determined by our experiments, to a previously reported data set^30^. The sequences were aligned using ClustalW^47^ and the results were manually checked to avoid improper gaps and/or shifts in the TM parts. The aligned sequences were then used for ML-based modeling.

To collect microbial rhodopsin genes for the training data set, BR^48^ and heliorhodopsin 48C12^49^ sequences were used as queries for searching homologous amino acid sequences in NCBI non-redundant protein sequences and metagenomic proteins^31^ and the *Tara* Oceans microbiome and virome database^32^. Protein BLAST (blastp)^37^ was used for the homology search, with the threshold E-value set at <10 by default, and sequences with >180 amino acid residues were collected. All sequences were aligned using ClustalW^47^. The highly diversified C-terminal 15-residue region behind the retinal binding Lys (BR Lys216) and long loop of HeR between helices A and B were removed from the sequences to avoid unnecessary gaps in the alignment. The successful alignment of the TM helical regions, especially the 3rd and 7th helices, was checked manually. The phylogenic tree was drawn using the neighbor-joining method^50^, and the microbial rhodopsin subfamilies were categorized based on the phylogenetic distances, as reported previously^38^. Based on the phylogenetic tree, 3022 putative ion-pumping rhodopsin genes from bacterial and archaeal origins were extracted, and their aligned sequences were used as the training data set for the prediction of *λ*_max_. The original training and test sets are provided in Supplementary Data 1 and Table 1, respectively, and the entire transformed datasets with physicochemical features (see Supplementary Data 2) are provided in Supplementary Data 7.

### ML modeling

Suppose that we have *K* pairs of an amino acid sequence and an absorption wavelength $\left\{ {\left( {{\boldsymbol{x}}^{\left( k \right)},\lambda _{{\mathrm{max}}}^{(k)}} \right)} \right\}_{k = 1}^K$, where ***x***^(*k*)^ ∈${\Bbb R}$ ^*MN*^ is the feature vector of the *k*-th amino acid sequence and $\lambda _{{\mathrm{max}}}^{(k)} \in {\Bbb R}$ is the absorption wavelength of the *k*-th rhodopsin protein. The least-absolute shrinkage selection operator (LASSO) is a standard regression model in which important regression coefficients can be automatically selected by the penalty on the absolute value of the coefficient, as follows:

$$\mathop {{\min }}\limits_{{\upmu },\,{\mathbf{\beta }}} \mathop {\sum }\limits_{k = 1}^K \left( {\lambda _{{\mathrm{max}}}^{\left( k \right)} - \mu - \mathop {\sum }\limits_{i = 1}^M \mathop {\sum }\limits_{j = 1}^N \beta _{i,j}x_{i,j}^{\left( k \right)}} \right)^2 +\,\gamma \mathop {\sum }\limits_{i = 1}^M \mathop {\sum }\limits_{j = 1}^N |\beta _{i,j}|,$$

where ${\boldsymbol{\beta}} \in {\Bbb R}^{MN}$ is a vector of *β*_*i,j*_ and *γ* > 0 is the regularization parameter. BLASSO is a Bayesian extension of LASSO for which the model is defined through the following random variables:

$$\lambda _{{\mathrm{max}}}^{\left( k \right)} \sim N\left( {\mu + {\boldsymbol{\beta}} ^{\it{ \top }}{\boldsymbol{x}}^{\left( k \right)},\sigma ^2} \right),{\boldsymbol{\beta}} \sim \pi \left( {{\boldsymbol{\beta}} |\sigma ^2} \right),$$

where *N*(*μ*,s^2^) is a Gaussian distribution with mean *μ* and variance *s*^2^, and $\pi \left( {{\boldsymbol{\beta}} \,|\,\sigma ^2} \right) = {\mathrm{{\Pi}}}_{i = 1}^M{\mathrm{{\Pi}}}_{j = 1}^N\frac{\gamma }{{2\surd \sigma ^2}}e^{ - \gamma |\beta _{i,j}|/\surd \sigma ^2}$ is the conditional Laplace prior. In this model, the maximum of the conditional distribution of the parameter ${\boldsymbol{\beta}} \mid \left\{ {\left( {{\boldsymbol{x}}^{\left( k \right)},\lambda _{{\mathrm{max}}}^{\left( k \right)}} \right)} \right\}_{k = 1}^K,\lambda ,\sigma$ is equivalent to the LASSO^51^ estimator. For *γ*, a hyper-prior is set through the gamma distribution prior on *γ*^2^, and the inverse gamma prior is assumed for *σ*^2^. For the computational details, see the original paper^36^. We used the “monomvn” package of R in our implementation. The prediction *f* (***x***) was sampled through the Gibbs sampler of ***β*** and *μ*. The number of samplings was set as *T* = 10,000 times. For each candidate ***x***, we approximately obtain ${\Bbb E}[{\mathrm{gain}}]$ by

$${\Bbb E}\left[ {{\mathrm{gain}}} \right] \approx \frac{1}{T}\mathop {\sum }\limits_{t = 1}^T \max \left( {\mu ^{\left( t \right)} + {\boldsymbol{\beta}} ^{\left( t \right){\it{ \top }}}{\boldsymbol{x}} - \lambda _{{\mathrm{base}}},0} \right),$$

where *μ*^(*t*)^ and ***β***^(*t*)^ are the *t*-th sampled parameters. The parameters of the trained model is provided in Supplementary Data 8.

### Protein expression

The synthesized genes of microbial rhodopsins codon-optimized for *E. coli* (Genscript, NJ) were incorporated into the multi-cloning site in the pET21a(+) vector (Novagen, Merck KGaA, Germany). The plasmids carrying the microbial rhodopsin genes were transformed into the *E. coli* C43(DE3) strain (Lucigen, WI). Protein expression was induced by 1 mM isopropyl β-d-1-thiogalactopyranoside (IPTG) in the presence of 10 μM all-*trans* retinal for 4 h.

### Measurement of the absorption spectra and *λ*_max_ of rhodopsins by bleaching with hydroxylamine

*E. coli* cells expressing rhodopsins were washed three times with a solution containing 100 mM NaCl and 50 mM Na_2_HPO_4_ (pH 7). The washed cells were treated with 1 mM lysozyme for 1 h and then disrupted by sonication for 5 min (VP-300N; TAITEC, Japan). To solubilize the rhodopsins, 3% *n*-dodecyl-d-maltoside (DDM, Anatrace, OH) was added, and the samples were stirred for overnight at 4 °C. The rhodopsins were bleached with 500 mM hydroxylamine and subjected to yellow light illumination (*λ* > 500 nm) from the output of a 1-kW tungsten−halogen projector lamp (Master HILUX-HR; Rikagaku) through colored glass (Y-52; AGC Techno Glass, Japan) and heat-absorbing filters (HAF-50S-15H; SIGMA KOKI, Japan). The absorption change upon bleaching was measured by a UV-visible spectrometer (V-730; JASCO, Japan).

### Ion-transport assay of rhodopsins in *E. coli* cells

To assay the ion-transport activity in *E. coli* cells, the cells carrying expressed rhodopsin were washed three times and resuspended in unbuffered 100 mM NaCl. A cell suspension of 7.5 mL at OD_660_ = 2 was placed in the dark in a glass cell at 20 °C and illuminated at *λ* > 500 nm from the output of a 1-kW tungsten–halogen projector lamp (Rikagaku, Japan) through a long-pass filter (Y-52; AGC Techno Glass, Japan) and a heat-absorbing filter (HAF-50S-50H; SIGMA KOKI, Japan). The light-induced pH changes were measured using a pH electrode (9618S-10D; HORIBA, Japan). All measurements were repeated under the same conditions after the addition of 10 μM CCCP.

### Imaging and electrophysiological assays

For heterologous expression in mammalian cultured cells, the synthesized rhodopsin genes were inserted into the cloning site between the CMV promoter and eYFP in phKR2-3.0-EYFP^52^ using EcoRI and BamHI. All experiments were carried out using ND7/23 cells, lined hybrid cells derived from neonatal rat dorsal root ganglion neurons fused with the mouse neuroblastoma, which were transfected with plasmids as previously described^53^. EYFP fluorescence (543 nm) in the ND7/23 cells expressing the rhodopsins were imaged under a confocal laser scanning microscopy (LSM510, Carl Zeiss, Oberkochen, Germany) at 512 × 512 pixels using a water-immersion objective (×63/0.95, Achroplan, Carl Zeiss) and Ar laser (514 nm). Currents were recorded using an EPC-8 amplifier (HEKA Electronic, Lambrecht, Germany) under a whole-cell patch clamp configuration while a 200 ms pulse illuminations at 549 ± 15 (nm, >90% of the maximum) and 28 mW‧mm^−2^ was given at 0.1 Hz using a SpectraX light engine (Lumencor Inc., Beaverton, OR). The internal pipette solution contained (in mM) 121.2 KOH, 90.9 glutamate, 5 Na_2_EGTA, 49.2 HEPES, 2.53 MgCl_2_, 2.5 MgATP, 0.0025 ATR (pH 7.4 adjusted with HCl). The extracellular Tyrode’s solution contained (in mM): 138 NaCl, 3 KCl, 2.5 CaCl_2_, 1 MgCl_2_, 10 HEPES, 4 NaOH, and 11 glucose (pH 7.4 adjusted with HCl).

### Statistical analysis

We assessed the effectiveness of the data-driven experimental design method by comparing it with random selection in terms of the proportions of observing red-shift gains in the selected rhodopsins. The statistical significance of the effectiveness was quantified by comparing the red-shift gain proportions 0.82 (=32/39, *p* = 7.025 × 10^−5^) with the probability of observing red-shift gains from randomly selected rhodopsins, i.e., 0.50, based on a binomial test. Since we set the base wavelength of each subfamily to the *λ*_max_ of rhodopsin which was studied in detail in previous work and equal or longer than the empirical median of the *λ*_max_ in each subfamily (Supplementary Fig. 2), it is reasonable to assume that the probability of observing red-shift gains from randomly selected rhodopsins must be smaller than or equal to 0.50. For statistical analysis of the ML model building and the evaluation of its performance, see the ML modeling section above.

### Reporting summary

Further information on research design is available in the Nature Research Reporting Summary linked to this article.

## Supplementary information

Supplementary Information

Description of Additional Supplementary Files

Supplementary Data 1

Supplementary Data 2

Supplementary Data 3

Supplementary Data 4

Supplementary Data 5

Supplementary Data 6

Supplementary Data 7

Supplementary Data 8

Supplementary Data 9

Reporting Summary

## Data Availability

All data shown in main figures were deposited in Supplementary Data 9. Data supporting the findings are available from the corresponding authors upon reasonable request.

## Code availability

The computational code of this manuscript is available at http://www-als.ics.nitech.ac.jp/~karasuyama/BLASSO-for-Rhodopsins/.
