# Author Correction: Exploration of natural red-shifted rhodopsins using a machine learning-based Bayesian experimental design

**DOI:** 10.1038/s42003-021-02090-5

**Published:** 2021-04-30

**Authors:** Keiichi Inoue, Masayuki Karasuyama, Ryoko Nakamura, Masae Konno, Daichi Yamada, Kentaro Mannen, Takashi Nagata, Yu Inatsu, Hiromu Yawo, Kei Yura, Oded Béjà, Hideki Kandori, Ichiro Takeuchi

**Affiliations:** 1 The Institute for Solid State Physics, The University of Tokyo, Kashiwa, Japan; 2 RIKEN Center for Advanced Intelligence Project, Tokyo, Japan; 3 Department of Life Science and Applied Chemistry, Nagoya Institute of Technology, Nagoya, Japan; 4 OptoBioTechnology Research Center, Nagoya Institute of Technology, Nagoya, Japan; 5 PRESTO, Japan Science and Technology Agency, Kawaguchi, Japan; 6 Department of Computer Science, Nagoya Institute of Technology, Nagoya, Japan; 7 Graduate School of Humanities and Sciences, Ochanomizu University, Tokyo, Japan; 8 Center for Interdisciplinary AI and Data Science, Ochanomizu University, Tokyo, Japan; 9 School of Advanced Science and Engineering, Waseda University, Tokyo, Japan; 10 Faculty of Biology, Technion-Israel Institute of Technology, Haifa, Israel

**Keywords:** Biophysics, Computational biology and bioinformatics, Biochemistry

Correction to: *Communications Biology* 10.1038/s42003-021-01878-9, published online 19 March 2021.

The original version of the Article contained an error in the second paragraph of the “Discussion” section. It was stated that “32 out of 39 microbial rhodopsins were found to have red-shifted absorption compared with the base wavelengths of each subfamily of microbial rhodopsins…”. However, after the publication, the authors became aware that the *λ*_max_ of BacHR from *Rubricoccus marinus* (Accession: WP 094550238.1) was previously reported to be 542 nm^1^, which is close to the value determined in this study (541 nm).

The original version of the Article also contained an error in the fourth paragraph of the “Discussion” section. It was stated that “Four rhodopsins showed red-shifted absorption ≥20 nm than the base wavelength, three of which showed light-driven ion transport function.”. After publication, the authors become aware that ion transport activities of BacHRs from *Rubricoccus marinus* (Accession: WP 094550238.1) and *Rubrivirga marina* (Accession: WP 095509924.1) were also previously reported^1^.
