# Supplementary material for: Exploration of natural red-shifted rhodopsins using a machine learning-based Bayesian experimental design
Source: Commun Biol. 2021 Mar 19;4:362. doi: 10.1038/s42003-021-01878-9 (PMC7979833; doi:10.1038/s42003-021-01878-9)
Supplement: Supplementary file 1 — Supplementary Information [file 42003_2021_1878_MOESM1_ESM.pdf]

Supplementary Information:

Exploration of natural red-shifted rhodopsins using a machine learning-based Bayesian experimental design

Keiichi Inoue*, Masayuki Karasuyama, Ryoko Nakamura, Masae Konno, Daichi Yamada, Kentaro Mannen, Takashi Nagata, Yu Inatsu, Hiromu Yawo, Kei Yura, Oded Béjà, Hideki Kandori, Ichiro Takeuchi*

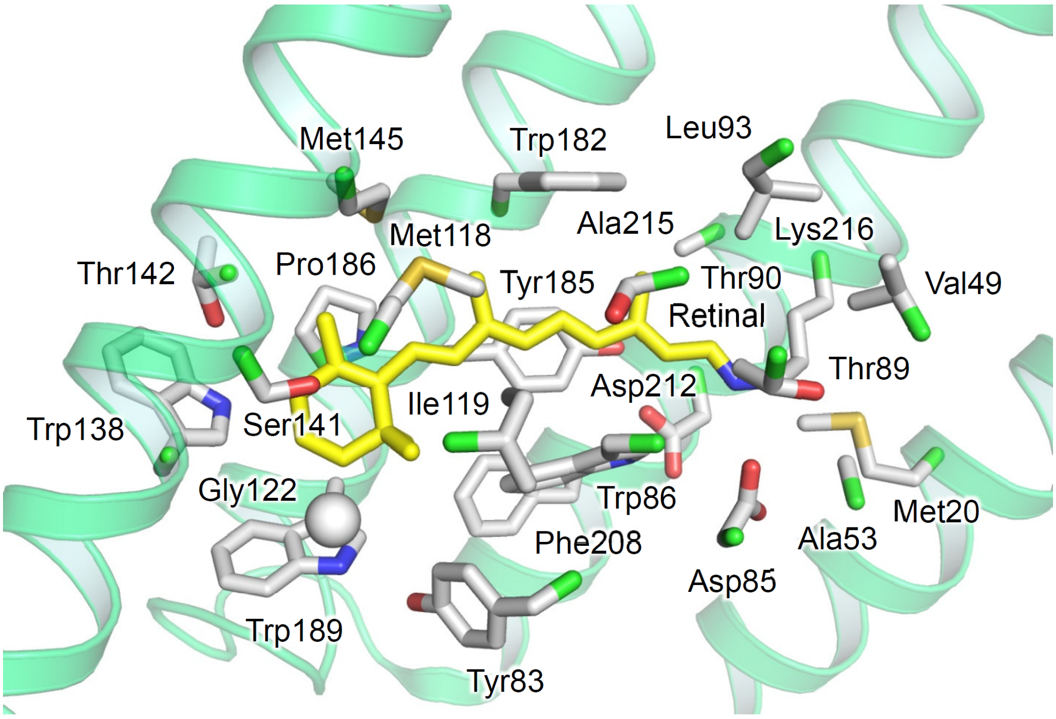

i	1	2	3	4	5	6	7	8
Residue in BR	Met20	Val49	Ala53	Tyr83	Asp85	Trp86	Thr89	Thr90

i	9	10	11	12	13	14	15	16
Residue in BR	Leu93	Met118	Ile119	Gly122	Trp138	Ser141	Thr142	Met145

i	17	18	19	20	21	22	23	24
Residue in BR	Trp182	Tyr185	Pro186	Trp189	Phe208	Asp212	Ala215	Lys216

Supplementary Figure 1. Amino acid residues around the retinal chromophore. The structure of the 24 amino acid residues around the retinal used in the current ML model in the X-ray crystallographic structure of BR (PDB ID: 1IW6 (Matsui et al. *J. Mol. Biol.* (2002) 324, pp. 469–481)). The Cα atom of Gly122 is shown as a white sphere. For clarity, the ribbon models of helices B, C, and E were omitted. The table lists the residue numbers and names of each residue in BR.

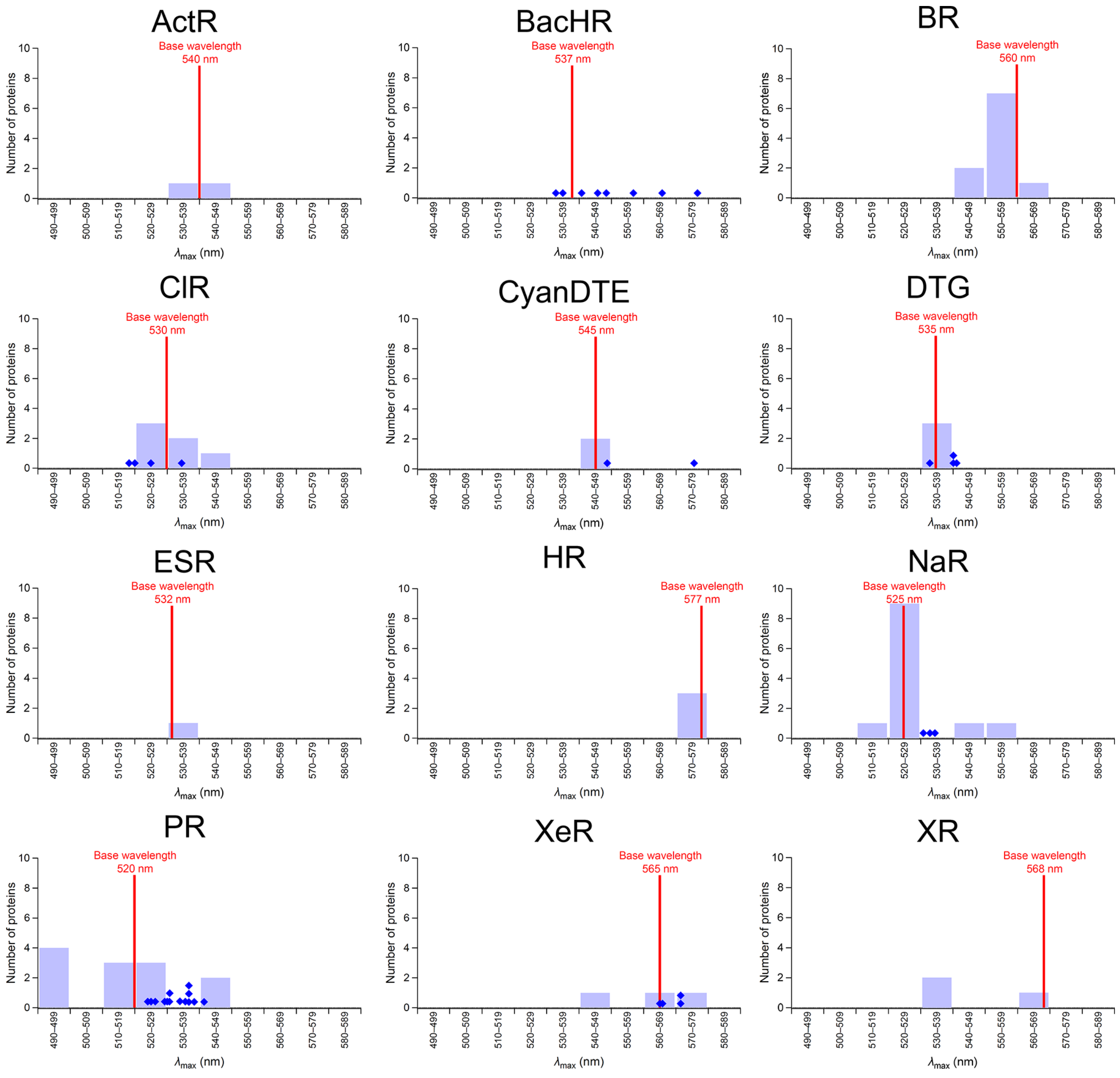

Supplementary Figure 2. Distribution of λ_{max} of wild-type microbial rhodopsins. Distribution of the number of microbial rhodopsins against their λ_{max} (blue columns) for ion-pumping rhodopsin subfamilies. The base wavelengths of each subfamily and experimentally observed wavelength obtained in this study were indicated by red lines and blue diamonds, respectively. Since λ_{max} of BacHR was not reported when we had constructed the training data, the distribution of the number of BacHRs against their λ_{max} cannot be shown. Then, λ_{max} of the first BacHR from cyanobacterium *Mastigocladopsis repens* was reported at 537 nm (Hasemi et al. *J. Biol. Chem.* (2016) 291, pp. 355–362)), and it was set to the base wavelength of BacHRs.
