# Supplementary material for: Exploration of natural red-shifted rhodopsins using a machine learning-based Bayesian experimental design
Source: Commun Biol. 2021 Mar 19;4:362. doi: 10.1038/s42003-021-01878-9 (PMC7979833; doi:10.1038/s42003-021-01878-9)
Supplement: Supplementary file 2 — Description of Additional Supplementary Files [file 42003_2021_1878_MOESM2_ESM.pdf]

Description of Additional Supplementary Files

File name: Supplemental Data 1
Description: Database for training the machine-learning model. Amino-acid sequences and the absorption wavelengths of 884 wild-type microbial rhodopsins and their variants are given.

File name: Supplementary Data 2
Description: The list of amino acid physicochemical features used as inputs in the ML model

File name: Supplementary Data 3
Description: The list of subfamilies and the base wavelengths of microbial rhodopsins.

File name: Supplementary Data 4
Description: The list of predicted red-shift gains of 3,022 genes. We selected 65 genes whose expected gains were > 10 nm. Note that, to synthesize a variety of genes, we randomly selected one representative among genes having identical amino acid sequences on the 24 retinal-surrounding residues.

File name: Supplementary Data 5
Description: The list of expected gains and observed gains of the selected 65 genes.

File name: Supplementary Data 6
Description: Amino acid residues at the color-tuning positions. The amino acid residues at the color-tuning positions corresponding to BR Leu93, Pro189, and Ala215.

File name: Supplementary Data 7
Description: The entire transformed datasets with physicochemical features.

File name: Supplementary Data 8
Description: The trained model parameters.

File name: Supplementary Data 9
Description: The source data underlying Fig. 4, 6a, 6b, 7a, and 7b.
